# [Retracted] Suppression of oxidative stress in endothelial progenitor cells promotes angiogenesis and improves cardiac function following myocardial infarction in diabetic mice

**DOI:** 10.3892/etm.2022.11626

**Published:** 2022-09-26

**Authors:** Peng Jin, Tao Li, Xueqi Li, Xinghua Shen, Yanru Zhao

Exp Ther Med 11:2163–2170, 2016; DOI: 10.3892/etm.2016.3236

Following the publication of this paper, it was drawn to the Editors’ attention by a concerned reader that certain of the B-scan ocular ultrasound images portraying cardiac function shown in Fig. 4A and B and Fig. 5A were strikingly similar to data appearing in different form in other articles published by different authors who were based at different institutions. Owing to the fact that the contentious data in the above article had already been published elsewhere prior to its submission to *Experimental and Therapeutic Medicine,* the Editor has decided that this paper should be retracted from the Journal. The authors were asked for an explanation to account for these concerns, but the Editorial Office did not receive a reply. The Editor apologizes to the readership for any inconvenience caused.

